# Technologies for whole‐cell modeling: Genome‐wide reconstruction of a cell in silico

**DOI:** 10.1111/dgd.12897

**Published:** 2023-11-08

**Authors:** Kazunari Kaizu, Koichi Takahashi

**Affiliations:** ^1^ RIKEN Center for Biosystems Dynamics Research Osaka Japan

**Keywords:** biological models, computer simulation, computer‐aided design, database, systems biology

## Abstract

With advances in high‐throughput, large‐scale in vivo measurement and genome modification techniques at the single‐nucleotide level, there is an increasing demand for the development of new technologies for the flexible design and control of cellular systems. Computer‐aided design is a powerful tool to design new cells. Whole‐cell modeling aims to integrate various cellular subsystems, determine their interactions and cooperative mechanisms, and predict comprehensive cellular behaviors by computational simulations on a genome‐wide scale. It has been applied to prokaryotes, yeasts, and higher eukaryotic cells, and utilized in a wide range of applications, including production of valuable substances, drug discovery, and controlled differentiation. Whole‐cell modeling, consisting of several thousand elements with diverse scales and properties, requires innovative model construction, simulation, and analysis techniques. Furthermore, whole‐cell modeling has been extended to multiple scales, including high‐resolution modeling at the single‐nucleotide and single‐amino acid levels and multicellular modeling of tissues and organs. This review presents an overview of the current state of whole‐cell modeling, discusses the novel computational and experimental technologies driving it, and introduces further developments toward multihierarchical modeling on a whole‐genome scale.

## Graphical abstract

There have been recent advances in an approach called “whole‐cell modeling,” which involves the modeling of cells on a whole‐genome scale. In this review, we present an overview of the current state of whole‐cell modeling and the computational and experimental technologies driving it.

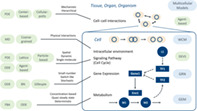

## 1 INTRODUCTION

Compared with the rapid development of techniques for cell measurement and synthesis, techniques for designing cells are still immature. Computer‐aided design is a powerful tool for the efficient design of cells, which is increasingly being employed in various applications. There have been recent advances in an approach called “whole‐cell modeling,” which involves the modeling of cells on a whole‐genome scale (Carrera & Covert, 2015; Goldberg et al., 2018; Karr, Takahashi, & Funahashi, 2015; Sanghvi et al., 2013; Tomita, 2001). In theoretical biology, models are built on a scale large enough to reproduce a specific biological phenomenon. Most models are therefore composed of a few elements. Since the rise of systems biology around the year 2000, models have been composed of relevant genes and molecules (Kitano, 2002a; Kitano, 2002b). However, the models have still been constructed focusing on a specific pathway or function of the cell. Many of these models consist of dozens of genes based on advances in genetics and omics methodologies. These models are based on the concept that only those genes associated with the specific cellular function can be isolated from the effects of other genes and cellular functions according to differences in time and spatial scales. However, such scale separation is not always possible because real cells are small in volume and multiple biological processes occur simultaneously. For example, in cells growing on limited resources such as prokaryotes, elevated expression of only a single gene can inhibit the expression of many other genes involved in different cellular functions and have a significant effect on metabolism (Choi et al., 2008). Therefore, to understand the interactions between multiple cellular functions, whole‐cell modeling constructs a model consisting of hundreds to thousands of genes and molecules. The whole‐cell model integrates multiple pathways and aims to comprehensively reproduce multiple cellular functions.

Attempts at modeling a whole cell were initially performed in mathematical biology, but most only modeled cell proliferation as a cellular function (Shuler et al., 1979). Pioneering work involved the whole‐cell modeling of a hypothetical self‐living cell, which was published in 1999 (Takahashi et al., 2002; Tomita et al., 1999). This work presented a model consisting of a gene expression system and multiple metabolic systems with a minimal complement of 127 genes based on *Mycoplasma genitalium*. The model achieved the minimum intracellular metabolism required for survival by uptake of nutrient sources, such as glucose, from the medium by phosphotransferases and synthesis of ATP by catabolism to lactic acid through glycolysis and fermentation. In this model, the enzymes and other proteins required for metabolism are synthesized by transcription and translation from their respective genes and degraded. In this study, a whole cell—albeit a hypothetical cell—was modeled at the genetic level. However, while this model was self‐sustaining, it did not possess the ability to proliferate, including DNA replication and progression through the cell cycle.

**FIGURE 1:**
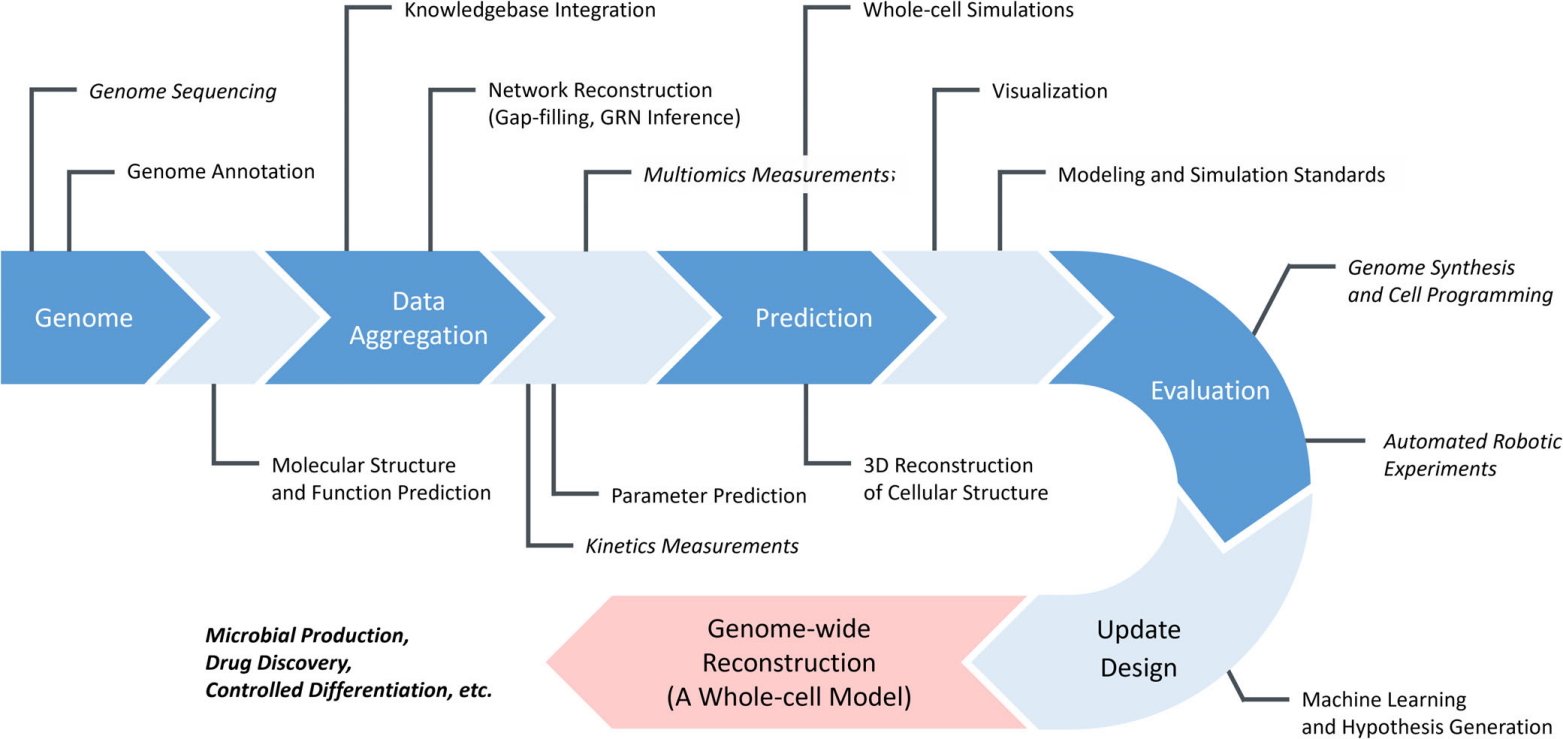
A brief overview of technologies for whole‐cell modeling. Whole‐cell modeling requires a variety of technologies across both experimental and computational biology, and these processes need to be repeated for practical modeling. Experimental technologies are indicated in italic.

Whole‐genome scale quantitative data have become available with the rapid development of multiomics methods and technologies for obtaining single‐cell and single‐molecule kinetic measurements. Interpretation of these vast amounts of data requires an understanding of whole‐cell systems as well as partial pathways. In 2012, a group at Stanford University proposed a whole‐cell model of *M. genitalium* with 525 genes (Covert, 2014; Karr et al., 2012). This model has the ability to undergo genome replication and cell proliferation, together with gene expression and metabolism. Furthermore, a whole‐cell model of *Escherichia coli* with 1,214 genes (43% of the well‐annotated genes, or 28% of all genes) was published in 2020 (Macklin et al., 2020). These whole‐cell models aim to reconstruct the function of every known molecule and reproduce the various observed cell behaviors, including multiomics data simultaneously. Genome‐scale metabolic modeling of yeast continues to be developed and extended to whole‐cell modeling (WCM) in a manner open to the scientific community (Dobson et al., 2010; Elsemman et al., 2022; Heavner et al., 2012, 2013; Lu et al., 2019). In 2019, the genome‐scale metabolic model (GEM) of budding yeast Yeast8, consisting of 2,680 metabolites, 1,133 genes, and 3,949 reactions, was published and used to develop the derived models for 1,011 different yeast strains (Lu et al., 2019). In addition to these uniform well‐stirred models, a three‐dimensional spatial whole‐cell model of JCVI–syn3A, a synthetic bacterium with 493 genes, has been constructed (Thornburg et al., 2019, 2022). For mammalian cells, whole‐cell modeling of human and cancer cells is underway (Münzner et al., 2019; Szigeti et al., 2018). Whole‐cell models in model organisms are becoming commonly available (Karr et al., 2013). They integrate multimodal data and knowledge about the subject organism, enabling predictions that are quantitatively comparable to a wide range of measurements (Carrera & Covert, 2015). By comparing these predictions with experimental measurements, we can test our knowledge and hypotheses, leading to new discoveries. Recently, whole‐cell models are also used in engineering for bioprocess optimization, for example, biomass and ethanol production, synthetic cell design (e.g., biosensors and minimal genomes), and clinical applications (e.g., drug design and delivery) (Chen et al., 2019; Gurdo et al., 2023; Marucci et al., 2020; Metzcar et al., 2019; Noll & Henkel, 2020). The following sections present novel technologies underpinning whole‐cell modeling and expected applications (Figure 1).

## 2 SIMULATION TECHNOLOGIES UNDERPINNING WHOLE‐CELL MODELING

**FIGURE 2:**
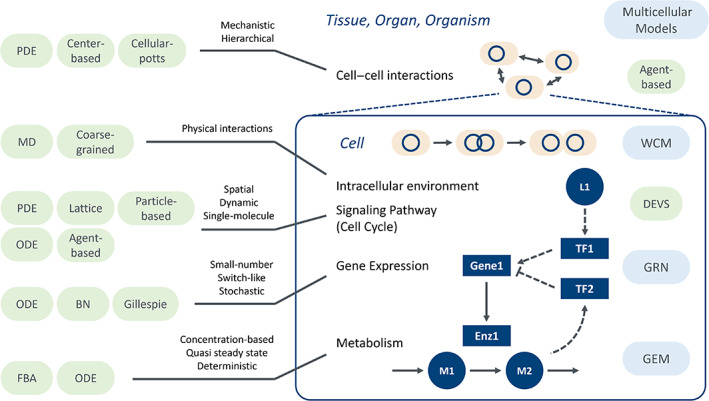
Submodels comprising the whole‐cell model and their requirements. Whole‐cell models consist of cellular pathways with different characteristics and require the integration of appropriate computational techniques. Representative computational techniques (green‐rounded rectangles) are shown corresponding to each cell function. Domain‐specific models are shown on the right (blue‐rounded rectangles).

Whole‐cell modeling involves many more components than conventional models, and the computational methods vary (Figure 2; Table S1). We have developed the E‐Cell system, software that allows a wide variety of simulation techniques and their combinations to provide an integrated platform for cell modeling, simulation, and analysis (Kaizu et al., 2020; Takahashi et al., 2004; Tomita et al., 1999).

The ordinary differential equation (ODE) is one of the most common descriptive methods in cell simulation. The first hypothetical self‐sustaining cell model was also described as ODEs. The ODE is a very effective method for reproducing the temporal changes in abundances of molecules in a cell governed by a biochemical reaction network, and has been applied to various systems, from metabolism to gene expression and signal transduction (Khodayari & Maranas, 2016; Kurata, 2021). However, it has several limitations: the number of molecules in the cell, which is by nature discrete, is treated as a continuous variable; it contains a large number of unknown parameters; and it cannot reproduce the behavior of molecules that do not satisfy the well‐stirred condition in a cell. First, most ODE models are based on averaged measurements of bulk cultures of cells in different states. However, in prokaryotic cells, the average number of molecules in a single cell is generally less than one, and the stochastic behavior of such a few molecules has a significant impact on cellular behavior (Choi et al., 2008; Taniguchi et al., 2010). In addition, biological phenomena utilizing such noise from a small number of molecules have been reported (McAdams & Arkin, 1999; Raj & van Oudenaarden, 2008; Raser & O'Shea, 2005). Second, enzymatic reaction equations require multiple kinetic parameters for each reaction, and these parameters are often unknown and must be estimated from measurement data (Karr, Williams, et al., 2015; Babtie & Stumpf, 2017). Parameter optimization in a nonlinear system is feasible for small‐scale models, but is challenging for ODEs with hundreds to thousands of dimensions. Furthermore, the distribution of molecules is not uniform in higher eukaryotic cells, which are spatially segmented by intracellular organelles (Thul et al., 2017). Even in prokaryotic cells, the distribution is nonuniform around the plasma membrane and genomic regions (Sanamrad et al., 2014). These nonuniform molecular distributions are often tightly related to cellular functions. However, it is difficult to capture the effects of molecular positions on reaction kinetics in ODEs.

The Gillespie method is a widely used algorithm for discrete and stochastic simulations that can capture the effects of intrinsic noise in biological systems (Gibson & Bruck, 2000; Gillespie, 1976, 1977). The algorithm simulates individual reaction events according to kinetic rates and the number of reactant molecules, and allows sampling from probability distributions governed by master equations. In contrast to the deterministic ODE model, the stochastic model can represent cellular variations in a population. However, the simulation takes a long time per run, especially for large numbers of molecules or fast reactions. In addition, due to the stochastic nature of the process, several attempts are required to capture the statistical behavior.

Metabolic flux analysis (FBA) is a method has been used very successfully for modeling metabolic systems (Orth et al., 2010; Palsson, 2000; Varma & Palsson, 1994a). FBA algebraically determines reaction rates from the fluxes of uptake and efflux of metabolites. Therefore, the model can be constructed from the reaction network without estimation of kinetic parameters. FBA models of whole metabolic pathways have been made in various organisms, including *E. coli*, budding yeast, and humans (Gu et al., 2019; Lu et al., 2019; Norsigian et al., 2020; Passi et al., 2021; Yilmaz & Walhout, 2017). For example, a model consisting of approximately 1,200 metabolites and 2,700 reactions regulated by 1,515 genes has been reported for *E. coli* (Monk et al., 2017). The FBA models assume a steady state, and therefore cannot reproduce temporal changes in the number of molecules. However, various extensions of FBA have been developed, such as the incorporation of gene expression regulation and combination of FBA with ODEs for temporal dynamics (Chandrasekaran & Price, 2010; Covert et al., 2008; He et al., 2016; Lu et al., 2022; Mahadevan et al., 2002; O'Brien et al., 2013; Varma & Palsson, 1994b). The whole‐cell model of *M. genitalium* described in 2012 also used FBA for the metabolic pathways (Karr et al., 2012). FBA is an effective method for whole‐cell modeling as well as metabolic engineering.

On the other hand, the Boolean network (BN) is a widely used method for representing and evaluating gene regulatory networks (GRNs) (Kauffman, 1969; Thomas, 1973). GRNs can be experimentally approximated from transcriptome analysis, and the Boolean network models have many fewer parameters than ODEs (Liu et al., 2014; Naldi et al., 2015; Shlomi et al., 2007). However, they are suitable for qualitative modeling, but less so for quantitative modeling of molecular abundance and temporal changes (de Jong, 2002; Karlebach & Shamir, 2008; Le Novère, 2015).

Molecular heterogeneity is often observed within cells, but genome‐wide modeling rarely considers reaction–diffusion. Partial differential equations (PDEs) are a general way of extending ODEs into space (Brown & Kholodenko, 1999; Clairambault, 2013; Cowan et al., 2012; Slepchenko et al., 2003; Turing, 1952). In cell simulations, PDEs are often applied to represent small molecules and ions, but are not suitable for representing small amounts of molecules or slow diffusion.

The next subvolume method, a spatial extension of the Gillespie method, divides space into small boxes and calculates the reactions of molecules within each box and diffusion between boxes (Elf & Ehrenberg, 2004; Stundzia & Lumsden, 1996). This method is commonly applied to signal transduction, but has yet to be combined with whole‐cell modeling (Drawert et al., 2012; Fange et al., 2012; Hattne et al., 2005; Hepburn et al., 2012).

Agent‐based modeling is an approach for considering the individual locations and states of each molecule, in which each molecule is represented as a distinct “agent” with unique properties (Bonabeau, 2002; Gorochowski, 2016). In modeling, locations represent the compartments, such as the membrane, nucleus, or intracellular organelles, and the binding positions on the genome rather than the three‐dimensional coordinates. Agent‐based modeling enables the reconstruction of molecular movement and collision on genomic DNAs, such as RNA polymerase and the DNA replication machinery. Recent developments in methods for single‐molecule kinetics measurements have made it possible to determine precise parameters, such as RNA polymerase and ribosomes in vivo and to provide experimental data for agent‐based modeling (Elf & Barkefors, 2019; Funatsu et al., 1995; Liu & Tjian, 2018; Weiss, 2000; Xie et al., 2008).

Molecular dynamics (MD) simulation is a higher‐resolution spatial representation. For example, MD models can reproduce the prokaryotic intracellular environment from the atomic level based on the structure, number, and arrangement of molecules (Feig et al., 2015; Frembgen‐Kesner & Elcock, 2013; Maritan et al., 2022; McGuffee & Elcock, 2010; Ridgway et al., 2008). However, many technical problems remain to be solved, such as the enormous computing time required, the initial molecular structure, and the quantum mechanics of a chemical process.

As shown above, various computational techniques have been developed corresponding to biological phenomena in cell simulations (Figure 2). The whole‐cell model consists of several cellular functions, each of which requires a suitable method. The discrete event system specification (DEVS) is a description for simultaneously computing different data representations and multiple computational techniques while maintaining independence between them (Zeigler, 2000). DEVS treats each algorithm as an event, identifies the dependencies between events from the referenced internal states, and resolves the time evolution by computing each event sequentially. In a whole‐cell system with a mixture of different spatial and time scales, no single method can adequately describe them all (Karr, Takahashi, & Funahashi, 2015; Takahashi et al., 2005). To reconstruct a wide range of cellular functions in a single model, it is essential to develop theories and techniques for integrating multiple methods consistently (Takahashi et al., 2004). In addition, improved modeling and simulation standards are required to share these comprehensive models (Hucka et al., 2003; Waltemath et al., 2016; Shaikh et al., 2022).

## 3 HOW TO CONSTRUCT WHOLE‐CELL MODELS EFFICIENTLY AND SUSTAINABLY

Many challenges remain in constructing whole‐cell models. Conventionally, researchers manually constructed cellular models based on information from the literature. The construction of models with a few to several dozen variables reproducing a single cellular function is feasible by a few groups with a deep understanding of the domain knowledge. Whole‐cell models, however, consist of thousands of variables and require a wide range of experimental data to be reproduced. In addition, the model contains many unknown parameters and diverse types of parameters, ranging from metabolic enzymes to regulation of gene expression (Karr, Williams, et al., 2015). It is difficult for a single researcher to understand and integrate all of the information behind a cell. Therefore, in whole‐cell modeling, it is essential to develop techniques to assist in the construction of models.

A portion of the parameters involved in whole‐cell modeling have been measured experimentally. For example, transcript, protein, and metabolite levels are estimated using transcriptomic, proteomic, and metabolomic analyses. Next‐generation sequencing and fluorescence imaging technologies have also been developed to quantify amounts of gene product in a single cell in vivo (Gorochowski et al., 2019; Lahtvee et al., 2017; Taniguchi et al., 2010). On the other hand, enzymatic kinetic parameters, such as the reaction activity *k*_cat_ and binding constant *K*_m_, have been determined in vitro by biochemical assays and stored in databases for each reaction (Chang et al., 2021; Wittig et al., 2012). However, these parameters were reported to differ between in vitro and in vivo conditions.

Single‐molecule measurement techniques have made it possible to precisely measure and model the kinetics of transcription, translation, replication, and degradation in vivo by fluorescence imaging of molecular machinery, such as RNA polymerase and ribosomes (Cai et al., 2006; English et al., 2006; Yu et al., 2006). It also enables the accurate quantification and estimation of the reaction–diffusion system in signaling pathways around the cellular membrane (Hiroshima et al., 2012; Sako et al., 2000). However, despite the successful applications in some subsystems, these measurements are limited to small scales and have yet to yield comprehensive genome‐wide measurements. For gene expression, the response curves have been experimentally measured for transcriptional regulatory sequences utilized in synthetic biology (Jones et al., 2022; Nielsen et al., 2016; Stanton et al., 2014). More recently, deep convolutional neural networks (CNNs) have been applied to predict expression levels from DNA sequences trained using transcriptome data with random sequences (de Boer et al., 2020; Vaishnav et al., 2022). These techniques can be expanded to comprehensive measurements on a genome‐wide scale.

Experimentally determined parameters have been separated for each category and stored in various databases. Recently, a database integrating these various experimental datasets has been published with the aim of whole‐cell modeling (Roth et al., 2021). A software platform has also been developed to construct cellular models in association with multiple databases (Bachman et al., 2022; Gyori et al., 2017).

As in the case of small‐scale models, whole‐cell models have also been constructed manually. However, new findings continue to be accumulated, and it is difficult to continually update large‐scale models manually. Therefore, technologies to automate construction are indispensable.

For example, the genome‐based modeling (GEM) system automatically prototypes cell‐scale metabolic simulation models from genome sequences and publicly available biological information (Arakawa et al., 2006). The system first predicts enzyme genes from genome sequences, collects parameters of their reactions from multiple enzyme and pathway databases, and automatically integrates them to build dynamic metabolic models. The system successfully generated metabolic models for 90 bacterial species. For example, the model of *E. coli* generated by the system consists of 1,195 metabolites, 968 reactions, and 835 enzymes. In addition, a new method has been developed to predict metabolic parameters that are unavailable in public databases (Chakrabarti et al., 2013; Smallbone & Mendes, 2013). This method can estimate unknown parameters from the fluxes determined by FBA and achieve a stable steady state. It was applied to genome‐wide models of *E. coli* with 402 reactions and 399 variables, and of budding yeast with 303 reactions and 282 variables. Furthermore, there is a technique for comprehensively extracting *k*_cat_ values for the maximal catalytic rates of enzymes from proteome data and fluxes under a variety of conditions (Davidi et al., 2016). More recently, a pipeline has been developed to predict *k*_cat_ by deep learning from enzyme sequences and substrate chemical formulas, and can construct genome‐scale metabolic models using Bayesian inference (Li et al., 2022). As described above, in metabolic engineering, the use of genome‐scale models is already essential for the quantification of enzyme kinetics.

Whole‐cell simulations produce considerable results in various data structures, making it difficult to obtain a complete picture. Furthermore, as whole‐cell models do not focus exclusively on specific cellular functions, finding new biological insights, such as significant changes and interactions, in the vast amount of simulation data is essential. Therefore, in addition to the model construction methods, techniques for visualization of whole‐cell models are critical to facilitate our understanding of the computational results. For example, in the whole‐cell model of *M. genitalium*, a digital dashboard composed of a wide variety of visual components was developed to browse results for cell proliferation, chromosomes, DNA‐binding molecules, metabolism, genome replication, and the total amounts of proteins and RNAs (Lee et al., 2013).

In whole‐cell simulations integrating multiple intracellular systems, it is also essential to display the interactions between subsystems. Transomics analysis is a technique for reconstructing global interaction networks between omics layers from multiomics data (Yugi et al., 2016; Zhou & Xia, 2018). The visualization of transomics analysis renders the networks of each omics layer in three‐dimensional space, helping to provide an understanding of the temporal changes in interregulation between gene expression, signaling, and metabolic subsystems. Furthermore, interactive environments connecting multiple researchers are required for iterative data exploration and collaborative construction of whole‐cell models (Cokelaer et al., 2015; Karr et al., 2013; Karr et al., 2014; Thiele et al., 2013; Waltemath et al., 2016). More recently, cross‐reality technologies, such as virtual reality (VR) and augmented reality (AR), have been developed and shown to be effective platforms for visualizing comprehensive data and models (Blanc et al., 2020; Lau et al., 2022; Legetth et al., 2021; Pirch et al., 2021; Zhang et al., 2019).

## 4 HOW WILL WHOLE‐CELL MODELING ADVANCE NEXT?

Whole‐cell modeling is increasingly becoming realistic and feasible due to developments in measurement techniques and improved computational performance. In recent years, innovative machine learning techniques have led to new approaches to whole‐cell modeling. In contrast to bottom‐up modeling based on molecular interaction networks, a predictive model for genome‐wide phenotypes of budding yeast has been constructed based on deep learning. DCell, trained on millions of budding yeast genotypes, can predict the cell growth rates of various mutants (Ma et al., 2018). In addition, 2,527 hierarchical structures of subsystems based on biological ontologies are embedded in the deep learning model, called a visible neural network (VNN). It visualizes the activities of subsystems in the cell and facilitates interpretation of the relationship between the phenotype and subsystems. Homogeneous and exhaustive experimental data are essential for this deep learning‐based phenotypic prediction. DCell is based on 23 million phenotypic data obtained by synthetic genetic array (SGA) analysis, which automates the construction of combined mutant strains using a robotic system (Costanzo et al., 2010, 2016).

Toward whole‐cell modeling, not limited to approaches using deep learning, it is imperative to develop experimental automation with robotic systems that allow the collection of comprehensive and highly accurate experimental data. However, it is not realistic to exhaustively measure all experimental targets in some experiments, such as whole‐genome scale combinations or phenotypic variations at single‐nucleotide level differences. For such experiments, a novel experimental technique to reduce the number of measurements is required. For example, the nonbiased random synthesis of promoter sequences and measurement of gene expression provide feasible predictions of promoter activity at the single‐nucleotide level by machine (Vaishnav et al., 2022). In addition, active learning combining robot experiments with AI inference can select prospective experimental targets and achieve highly efficient and low‐cost measurements (King et al., 2004, 2009; Rozanski et al., 2015; Sverchkov & Craven, 2017).

Whole‐cell modeling was first directed toward unicellular organisms, such as *E. coli* and budding yeasts. Therefore, its main applications were in producing useful substances and discovering drugs, such as antimicrobials (Carrera & Covert, 2015; Macklin et al., 2014; Marucci et al., 2020; Sanghvi et al., 2013). Meanwhile, models of cultured human cells have also been constructed and applied to cell differentiation and the medical field (Szigeti et al., 2018). In addition, new techniques have been developed to extend these sophisticated models of single cells to cell populations and organs (Dada & Mendes, 2011; Ghaffarizadeh et al., 2018; McCulloch, 2016; Montagud et al., 2021). These simulation techniques integrate a hierarchy of subcellular and cell population dynamics, modeling various biological phenomena, such as tumorigenesis in cancer and cellular rearrangements in development. In cancer biology, cell‐based computational models have been applied to investigate how the behavior of individual cells affects the clinical progression of tumors (Metzcar et al., 2019). In cancer systems, heterogeneous cancer cells form tumors through interactions with other cells and the tissue microenvironment. In addition to single‐cell modeling, various computational techniques are required to simulate cell–cell interactions, cell morphology, cell division, and cell migration to assess the effects of drugs based on the mechanisms of tumor development (Osborne et al., 2017). Although the application of whole‐cell modeling to the tissue or organ scale is still limited, these techniques will provide a common basis for future multiscale whole‐cell modeling. Conversely, single‐molecule level simulation reproduces cellular dynamics from a hierarchy of single‐molecule or single‐atom scales (Andrews, 2017; Arjunan & Tomita, 2010; Hoffmann et al., 2019; Michalski & Loew, 2016; Sokolowski et al., 2019; van Zon & ten Wolde, 2005). The single‐molecule simulation technique extends the traditional agent‐based approach to calculate reactions in three‐dimensional space from molecular diffusion and incorporate molecular structure and conformational changes (Thornburg et al., 2022). As a further large‐scale hierarchical model, a digital twin of the immune system integrating multiscale biological data at the molecular, cellular, tissue, organ, and body levels for personalized medicine in humans is planned (Laubenbacher et al., 2022). There will be further development of next‐generation technologies for integrating multiscale hierarchical models, that is, whole‐organ and whole‐body modeling in the near future.

Genome editing technologies have progressed from traditional gene‐by‐gene modification to long DNA synthesis at single‐nucleotide resolution, and sophisticated cell synthesis techniques have enabled flexible control of artificial cellular compositions. Along with such advances in synthesis technology, to predict cellular dynamics from single‐nucleotide variations in the genome, it is necessary for whole‐cell modeling to incorporate different computational biology disciplines in a comprehensive manner. For example, systems biology can elucidate spatiotemporal dynamics of a cell from molecular interactions, bioinformatics analyzes genome sequence and multiomics data, and structural biology reproduces the molecular structure and function from amino acid sequences. As mentioned above, the experimental biological data and knowledge behind whole‐cell modeling are also diverse, including genetics, biochemistry, molecular biology, cell biology, biophysics, and synthetic biology. Whole‐cell modeling integrates the current understanding of the target cell in an objective manner as a mathematical model. Accordingly, whole‐cell modeling provides a platform for mutual understanding and unification among various hierarchies and disciplines. It is not just a matter of computational biology, but one of the ultimate goals in biology.

## Supporting information


**TABLE S1.** Models representing whole‐cell modeling approaches. Models for which no implementation is shown in the original paper are marked with ‘‐’ in the ‘Methods’ column. WCM = Whole‐cell modeling; GEM = Genome‐scale metabolic model; ODE = Ordinary differential equation; PDE = Partial differential equation; FBA = Flux balance analysis; RDME = A reaction–diffusion master equation; GRN = Gene regulatory network; BN = Boolean Network.
