# Supplementary material for: Technologies for whole‐cell modeling: Genome‐wide reconstruction of a cell in silico
Source: Dev Growth Differ. 2023 Nov 8;65(9):554–64. doi: 10.1111/dgd.12897 (PMC11520977; doi:10.1111/dgd.12897)
Supplement: Supplementary file 1 — TABLE S1. Models representing whole‐cell modeling approaches. Models for which no implementation is shown in the original paper are marked with ‘‐’ in the ‘Methods’ column. WCM = Whole‐cell modeling; GEM = Genome‐scale metabolic model; ODE = Ordinary differential equation; PDE = Partial differential equation; FBA = Flux balance analysis; RDME = A reaction–diffusion master equation; GRN = Gene regulatory network; BN = Boolean Network. [file DGD-65-554-s001.docx]

| Model | Organism / Model category | Methods |
| --- | --- | --- |
| (Shuler et al., 1979) | *E. coli*; WCM | ODE |
| E-Cell1 (Tomita et al., 1999; Takahashi et al., 2002) | The self-sustainable cell based on *M. genitalium*; WCM | ODE |
| (Karr et al., 2012; Covert, 2014) | *M. genitalium*; WCM | ODE, Agent-based, FBA |
| (Macklin et al., 2020) | *E. coli*; WCM | ODE, Agent-based |
| (Szigeti et al., 2018) | Human cells; WCM | - |
| DCell (Ma et al., 2018) | Budding yeast; WCM | Deep-leaning |
| (Thornburg et al., 2022) | JCVI-syn3A; WCM | ODE, Subvolume (RDME) |
| (Münzner et al., 2019) | Budding yeast; Cell Cycle | Boolean |
| *i*ML1515 (Monk et al., 2017) | *E. coli*; GEM | FBA |
| Yeast8 (Lu et al., 2019) | Budding yeast; GEM | FBA |
| k-ecoli457 (Khodayari & Maranas, 2016) | *E. coli*; GEM | ODE |
| (Kurata, 2021) | Human cells; GEM | ODE |
| (Chandrasekaran & Price, 2010) | *E. coli*, *M. tuberculosis*; GEM | FBA, GRN (Probabilistic) |
| (O’Brien et al., 2013) | *E. coli*; GEM | FBA (ME-Model) |
| (Elsemman et al., 2022) | Budding yeast; GEM | FBA, GRN (BN) |
| GEM System (Arakawa et al., 2006) | *E. coli*, etc.; GEM | ODE |
| (Smallbone & Mendes, 2013) | *E. coli*, Budding yeast; GEM | ODE |
| ORACLE (Chakrabarti et al., 2013) | *E. coli*; GEM | ODE |
| DL-ecGEM (Li et al., 2022) | Budding yeast, etc.; GEM | ODE |
| (Ridgway et al., 2008) | *E. coli*; Macromolecules | Coarse-grained (Monte-Carlo) |
| (McGuffee & Elcock, 2010) | *E. coli*; Macromolecules | Brownian Dynamics |
| (Feig et al., 2015) | *M. genitalium;* Macromolecules | Full atomistic |
| (Maritan et al., 2022) | *M. genitalium*; Macromolecules | 3D Structure |
| PhysiCell (Ghaffarizadeh et al., 2018) | Tumor; Multicellular | PDE, Agent-based |
| (Laubenbacher et al., 2022) | The human immune system; Digital twin | - |

Supplementary Table 1. Models representing whole-cell modeling approaches. Models for which no implementation is shown in the original paper are marked with '-' in the 'Methods' column. WCM = Whole-cell modeling; GEM = Genome-scale metabolic model; ODE = Ordinary differential equation; PDE = Partial differential equation; FBA = Flux balance analysis; RDME = A reaction-diffusion master equation; GRN = Gene regulatory network; BN = Boolean Network;

| Model | Target | Methods |
| --- | --- | --- |
| (Shuler et al., 1979) | Cell proliferation |  |
| E-Cell (Tomita et al., 1999; Takahashi et al., 2002) | The virtual cell based on *M. genitalium*; WCM | ODE |
| (Karr et al., 2012; Covert, 2014) | *M. genitalium*; WCM | ODE, Agent-based, FBA |
| (Macklin et al., 2020) | *E. coli*; WCM | ODE |
| (Szigeti et al., 2018) |  |  |
| (Elsemman et al., 2022) |  |  |
| (Münzner et al., 2019) |  |  |
| (Lu et al., 2022) |  |  |
| (Khodayari & Maranas, 2016) |  |  |
| (Kurata, 2021) |  |  |
| (Varma & Palsson, 1994a; Palsson, 2000; Orth et al., 2010) |  | FBA |
| (Monk et al., 2017) |  | FBA |
| (Varma & Palsson, 1994b; Mahadevan et al., 2002; Covert et al., 2008; Chandrasekaran & Price, 2010; O’Brien et al., 2013; He et al., 2016; Lu et al., 2022) |  | FBA |
| (Shlomi et al., 2007; G. Liu et al., 2014; Naldi et al., 2015) |  | GRN (Boolean Network) |
| (de Jong, 2002; Karlebach & Shamir, 2008; Le Le Novère, 2015) |  |  |
| (Turing, 1952; Brown & Kholodenko, 1999; Slepchenko et al., 2003; Cowan et al., 2012; Clairambault, 2013) |  | PDE |
| (Stundzia & Lumsden, 1996; Elf & Ehrenberg, 2004) |  |  |
| (Hattne et al., 2005; Fange et al., 2012; Drawert et al., 2012; Hepburn et al., 2012) |  |  |
| (Frembgen-Kesner & Elcock, 2013; Feig et al., 2015; Maritan et al., 2022) |  |  |
| (Roth et al., 2021) |  |  |
| (Gyori et al., 2017; Bachman et al., 2022) |  |  |
| (Arakawa et al., 2006) |  |  |
| (Smallbone & Mendes, 2013; Chakrabarti et al., 2013) |  |  |
| (Davidi et al., 2016) |  |  |
| (Li et al., 2002) |  |  |
| DCell (Ma et al., 2018) | Budding yeast; WCM | Deep-leaning |
| (Dada & Mendes, 2011; Ghaffarizadeh et al., 2018; McCulloch, 2016; Montagud et al., 2021) |  |  |
| (Laubenbacher et al., 2022) |  |  |
| (Bicount & Field, 1996) | *E. coli*; Macromolecules | Coarse-grained |
